# Complex Problem Solving and Its Position in the Wider Realm of the Human Intellect

**DOI:** 10.3390/jintelligence6010005

**Published:** 2018-01-25

**Authors:** Samuel Greiff, Ronny Scherer

**Affiliations:** 1 Cognitive Science & Assessment, University of Luxembourg, 4366 Esch-sur-Alzette, Luxembourg; 2 Centre for Educational Measurement at the University of Oslo (CEMO), 0373 Oslo, Norway; ronny.scherer@cemo.uio.no

**Keywords:** complex problem solving, intelligence, human intellect

We cannot solve our problems with the same thinking we used when we created them.

—Albert Einstein (1879–1955)

Over the last century, a wealth of studies has accumulated that provides evidence for the paramount relevance of general mental ability or *g* as an overarching indicator of the human intellect for virtually any indicator of life success [1]. For instance, Deary, Strand, Smith, and Fernandes (2007) report a correlation of 0.81 between a latent *g*-factor score and a latent factor of educational achievement in a prospective study of 70,000 secondary students over five years [2]. In fact, there are many other studies that support this pattern of results. Also, comprehensive theories such as the Cattell-Horn-Carroll-theory (CHC-theory) often contain *g* as the most general (and most relevant) factor at the apex, but they usually include additional factors that are sometimes located on the same level as *g* and sometimes located on conceptually lower or more specific levels [3]. For instance, CHC-theory assumes a broad *g*-factor on the top (stratum III), which is further differentiated into lower-level and more specific cognitive abilities (stratum II and stratum I) [3].

The type, number, and nature of these additional factors have been subject of intensive and controversial scientific debate, and CHC-theory is still experiencing extensions, particularly with regard to stratum II and stratum III abilities [4]. Some of the efforts that focus on extensions of existing theories of the human intellect are accompanied by general criticisms on the abstract nature of standard tests of *g*, such as Raven’s Advanced Progressive Matrices [5]. These tests are, according to their critics, largely out of touch with reality and have limited real-world relevance. Moreover, there is a need for measures that capture certain aspects of the human intellect more comprehensively in addition to and beyond *g* [6,7].

As a response to this need, complex problem solving (CPS) has received considerable attention over the last couple of years as one potential extension of current theories. Both its role within the nexus of other cognitive abilities as well as its assessment are currently under intensive scientific investigation [8,9,10,11]. The core of this special issue is to shed further light on the role of CPS within the broad field of research on the human intellect.

When first research reports on CPS emerged in the 1970s, it was the complex and highly face-valid nature of the problem scenarios used in this line of research, in which participants had to explore complex systems and work through environments that tried to mimic real-life scenarios, that was considered important and a viable alternative to or augmentation of existing measures of intelligence [7]. However, the hope put into CPS diminished rather quickly because the conceptual delineation between CPS and *g* was difficult to establish and to substantiate empirically. Conceptual fuzziness and issues in the assessment and the scoring of complex scenarios hampered a thorough understanding of CPS as a construct, its assessment, and its utility for human performance in general [12,13,14]. It was only recently that CPS was re-discovered and that scientific interest in CPS was sparked once again. A number of reasons contributed to this development, but out of those three are arguably most noteworthy: firstly, theories on the nature of the human intellect continue to experience extensions. For instance, several new stratum II abilities have emerged as relevant augmentations of CHC-theory and this ongoing search also includes higher-order thinking skills such as CPS. Secondly, one of the core reasons why interest in CPS declined towards the end of the 20th century was the persistent problems with regard to the measurement of the multi-faceted construct of CPS. Recently developed assessment approaches have solved some of the former methodological issues and, thus, now allow for a renewed and intensified content-related debate on the nature of CPS. Thirdly and from a more political stance, changes in the way educational systems need to prepare students for the challenges they face later in life have contributed to the interest in CPS. More specifically, there is a development away from the learning and instruction of rote knowledge and a move toward the teaching of more cross-curricular skills such as CPS. In fact, CPS was recently included as a critical 21st century skill within educational large-scale assessments and across a number of diverse countries [15].

While interest in CPS has risen anew, several questions related to the nature of CPS as a cognitive ability, its assessment, and the practical relevance of CPS remain up to now unanswered. The aim of this special issue is therefore to contribute to the academic discussion surrounding CPS and, in doing so, to advance our knowledge in this vibrant field of research. This special issue entitled “Complex Problem Solving and its Position in the Wider Realm of the Human Intellect” in the *Journal of Intelligence* comprises several papers, some of them conceptual, some of them empirical, and some of them a mixture of both that employ different measures of CPS and that address a diversity of different research questions.

The first set of two papers in this special issue provides general insights into the concept of CPS and its underlying processes and discusses the role of CPS as part of broader, socially embedded human activities. In the first paper, Beckmann and Goode (this issue) propose a conceptually novel framework of “complexity” as an integral theoretical aspect of CPS and put it to an empirical test. Graesser, Kuo, and Liao (this issue) discuss the concept of “collaborative problem solving” as a theoretical construct that combines the CPS processes with human collaboration, and present the integration of collaborative problem solving into large-scale educational assessments.

The second set of three papers in this special issue discusses the specific relation of CPS to other concepts, partly from a theoretical and partly from an empirical perspective. Murphy (this issue) highlights the role of general (i.e., *g*) and specific abilities (e.g., CPS) in the prediction of job performance, and provides a historical overview of the “not-much-more-than-*g*-debate” along with some implications for concurrent CPS research. Kyllonen, Anguiano Carrasco, and Kell (this issue) tackle a similar issue. They examine the relation between CPS and fluid intelligence conceptually and empirically by reviewing different definitions of CPS and its underlying demands as well as the perceived relevance of CPS as compared to *g*. In a review and a substantial extension of three already published studies, Zech, Bühner, Kröner, Heene, and Hilbert (this issue) discuss apparently contradictory results on the relations among working memory, reasoning, and CPS. They conclude that the discrepancies in results between the different studies can be explained by differences in the symmetry level of abstraction in the measures as well as by differences in the content of the working memory and reasoning measures.

The final paper by Güss, Edelstein, Badibanga, and Bartow (this issue) in this special issue focuses on the empirical investigation of CPS processes. The authors compare experts and novices in a CPS business simulation and report how the groups differ not only in overall performance but also in their specific behaviors over the course of the simulation.

The special issue features diverse articles that are all related to the nature of CPS and how it is embedded within the wider realm of the human intellect. At the same time, it illustrates how difficult it is to investigate the position of CPS (across several measures) next to cognate cognitive abilities that describe human intellect (e.g., fluid intelligence, executive functions) and to establish a clear picture of the nomological network of CPS. Put differently, we as the guest editors of this special issue encourage more theory-guided research that establishes a direct link, for instance, between CHC-theory and CPS and its (possible) role as a stratum II ability.

At the same time, we encourage the integration of theoretical perspectives about where CPS can be located on the map of cognitive theories and assessment perspectives. More precisely, the difficulty to investigate the position of CPS next to other cognitive abilities may be at least partly due to the difficulty to obtain sufficient evidence for the construct validity of cognitive assessments. This issue not only requires researchers’ attention but may also facilitate the critical reflection of the processes underlying cognitive abilities. The current development to assess CPS and cognate constructs through computer-based test delivery (e.g., with the help of simulations or microworlds) creates unique opportunities to study these processes in depth.

Besides, the papers presented in this special issue highlight the importance of defining what determines and what is meant by the term “complexity”, the Big C in CPS. Of course, certain design characteristics of CPS tasks (e.g., the number of relations among variables) and the situational demands in CPS scenarios (e.g., individual vs. collaborative problem solving) contribute to complexity. At the same time, what creates complexity in problem-solving situations might, in fact, change over time—given the rapid advancement of technology, the availability of information, and the increasing exposure to problems that require more than just the application of routines. In other words, problems that are considered complex today might not be considered complex tomorrow. It is therefore necessary to understand how the human mind adapts to increasingly complex problem situations. Once again, this requires solid theoretical ground on what complexity is and how the human mind deals with it.

Irrespective of the specific theoretical setup, it becomes clear that much work remains to be done, and we as the guest editors of this special issue hope that the articles in this special issue inspire future research—on the human intellect and possibly even on CPS.
